# Ocular Duction Measurement Using Three Convolutional Neural Network Models: A Comparative Study

**DOI:** 10.7759/cureus.73985

**Published:** 2024-11-19

**Authors:** Suthicha Chuntranapaporn, Raveewan Choontanom, Worapot Srimanan

**Affiliations:** 1 Ophthalmology, Phramongkutklao Hospital, Bangkok, THA

**Keywords:** artificial intelligence, convolutional neural networks, measurement, ocular duction, ophthalmology

## Abstract

Objective: This study primarily aimed to compare the accuracy of three convolutional neural network (CNN) models in measuring the four positions of ocular duction. Further, it secondarily aimed to compare the accuracy of each CNN model in the training dataset versus the ophthalmologist measurements.

Methods: This study included 526 subjects aged over 18 who visited the ophthalmology outpatient department. Ocular images were captured using mobile phones in various gaze positions and stored anonymously as JPEG files. Ocular duction was measured by assessing corneal light reflex deviation from the central cornea. Ductions were classified into 30, 60, and 90 prism diopters (PD) and full ductions from the primary position. Three CNN models, MobileNet, ResNet, and EfficientNet, were used to classify ocular duction. Their predictive ability was evaluated using the area under the receiver operating characteristic (AUROC) curve. The dataset was divided into the training (2,001 images), evaluation (213 images), and testing (190 images) groups, which were reconstructed using the routine follow-up data of volunteers at the Ophthalmology Department of Phramongkutklao Hospital between February 2023 and June 2023.

Results: To evaluate the data, the MobileNet_V3_Large, ResNet101, and EfficientNet_B5 models were utilized to measure duction angles with the receiver operating characteristic (ROC) curves. The training times for MobileNet, ResNet, and EfficientNet were 5.54, 9.56, and 26.39 minutes, respectively. In the testing phase, MobileNet, ResNet, and EfficientNet were used to measure each duction position: 30 PD with corresponding ROC curve values of 0.77, 0.5, and 0.58; 60 PD with ROC curve values of 0.71, 0.83, and 0.81; 90 PD with ROC curve values of 0.7, 0.73, and 0.81; and full duction with ROC curve values of 0.91, 0.93, and 0.94, respectively. Analysis of variance revealed no significant difference in the mean AUROC curves among the models, yielding a p-value of 0.936. MobileNet has the narrowest confidence intervals for average prediction accuracy across three CNN models.

Conclusions: The three CNN models did not significantly differ in terms of efficacy in detecting various duction positions. However, MobileNet stands out, with a narrower confidence interval and shorter training time, which indicates its potential application.

## Introduction

In ophthalmology, ocular duction measurement can help understand eye muscle functionality and identify abnormalities including strabismus. Hence, it is essential for diagnosing and treating eye conditions [1]. Clinical ocular measurement methods vary from subjective clinician grading, which is dependent on individual expertise and, thus, subject to variability [2], to less commonly used objective techniques such as the application of a cervical range of motion device [3], Kestenbaum limbus test [4], laser pointer technique [5], and Goldmann perimetry technique [6-8]. The objective techniques have a complex setup and are, thus, time-consuming. Moreover, their use requires specialized skills. Hence, they are not commonly used. The photographic base was adapted to record and interpret ocular movements [9-12]. Nevertheless, manual measurement, which can introduce interobserver variability, is required.

Artificial intelligence (AI) in ophthalmology streamlines diagnostics, thereby facilitating a precise analysis of complex data such as retinal images and visual field test results [13,14]. This technology enhances accuracy, expedites diagnostic processes, and facilitates early pathology detection. Hence, it can be beneficial for ophthalmologists in streamlined workflows, particularly for conditions such as diabetic retinopathy and glaucoma [13,14]. Recent research has introduced AI-based photographic methods for detecting strabismus based on diverse reference techniques [15-17]. Deep learning-based image analysis categorizes eye versions in patients with strabismus [16] and differentiates ocular movements at various ages [17]. These advancements capitalize on convolutional neural networks (CNNs) [18] and other AI algorithms, thereby reinforcing diagnostic capabilities and enhancing management strategies for ocular anomalies.

A diverse range of CNN models for image analysis exhibit distinct characteristics related to accuracy, computational efficiency, and processing speed [18,19]. With unique designs and configurations, these models offer various precision levels, resource utilization, and temporal efficiencies in interpreting visual data. They have specific precision, resources, and time considerations in image analysis frameworks.

This study primarily aimed to analyze the precision of three distinct CNN models in quantifying four ocular duction ranges. In addition, it secondarily aimed to compare the accuracy of each model in the training dataset and the ocular measurements obtained by ophthalmologists. The four ranges of duction were measured based on corneal light reflex deviation from the central cornea, which were 30, 60, and 90 prism diopters (PD) and full of duction pass from the midline [20].

## Materials and methods

Study design and setting

An institutional-based cross-sectional prospective study was conducted on volunteers who attended the eye check-up programs at the Ophthalmology Outpatient Department of Phramongkutklao Hospital between December 1, 2022, and May 31, 2023. This study was conducted in accordance with the Declaration of Helsinki. The Institutional Review Board of the Royal Thai Army Medical Department approved the current study on February 6, 2023 (approval number: R121h/65).

Study population

Adult participants aged 18 years visiting the ophthalmology outpatient department were included in this study. The exclusion criteria were individuals with a previous history of ocular surgery, ocular defects, strabismus, neurological conditions affecting cranial nerves 3, 4, or 6, and ocular surface diseases. All participants provided informed consent for publication as their identities are discernible in the images, a mandatory prerequisite for study inclusion.

Data collection

**Figure 1:**
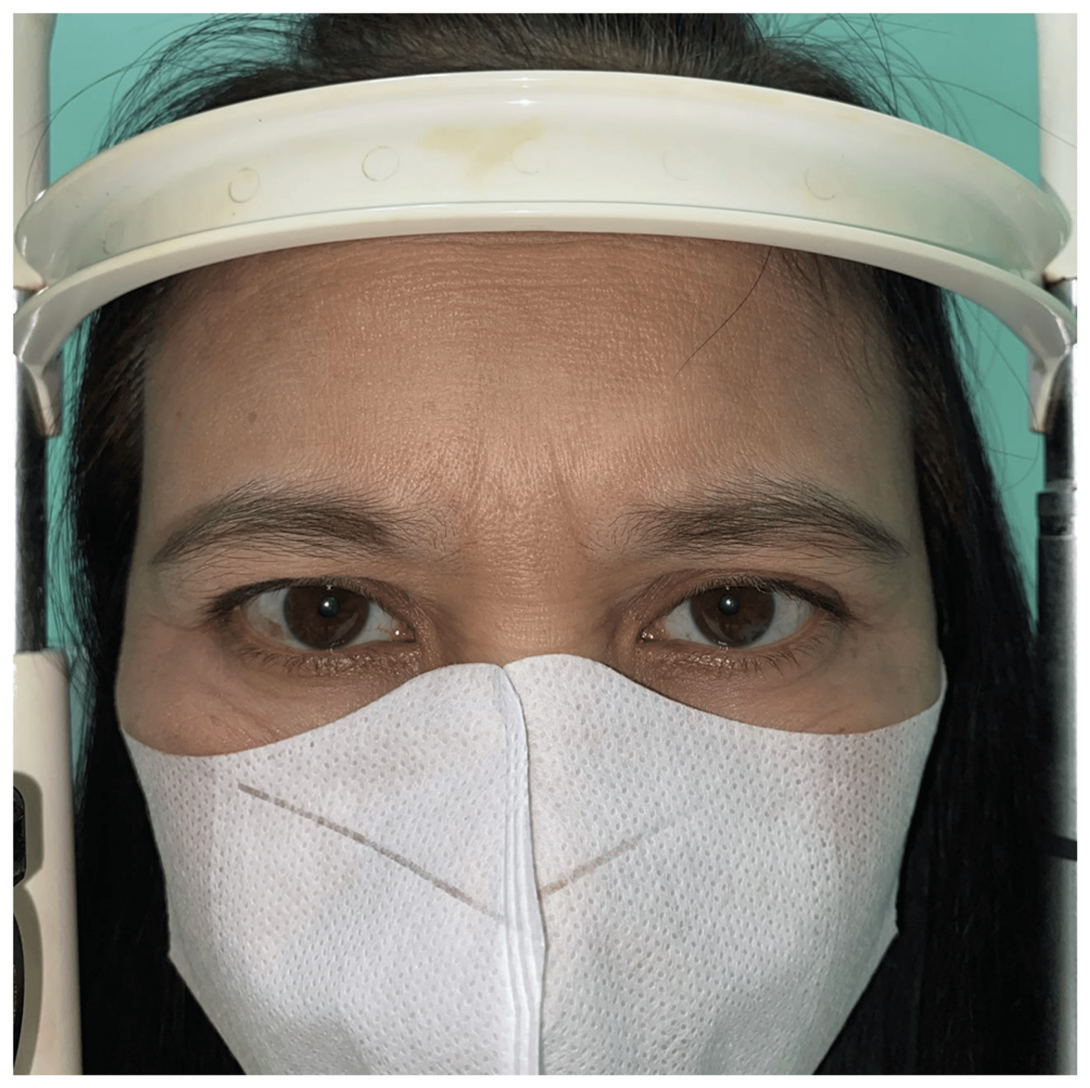
Patient's primary position when capturing eye images

The participants sat in a chair facing the slit lamp, with their chin and forehead resting on the support structure, as shown in Figure 1. This was the primary orientation. Each participant was instructed to move their eyes sequentially in four directions, left, right, up-gaze, and down-gaze, while an iPhone 11 camera captured consecutive images. The gaze direction was for taking images and detecting the corneal light reflex, which is not important in the interpretation of duction.

The distance between the smartphone and the participant was maintained at 14 inches. The images obtained using the smartphone were stored as JPEG files accompanied by date and numerical labels to ensure participant anonymity. To assess image quality, the researchers evaluated pixel-specific details such as color, shadows, and contrast, representing the image quality assessment.

The amount of corneal light reflex deviating from the central cornea was a measurement technique for recording the duction range and a fundamental ground truth reference for interpretation [20].

**Figure 2:**
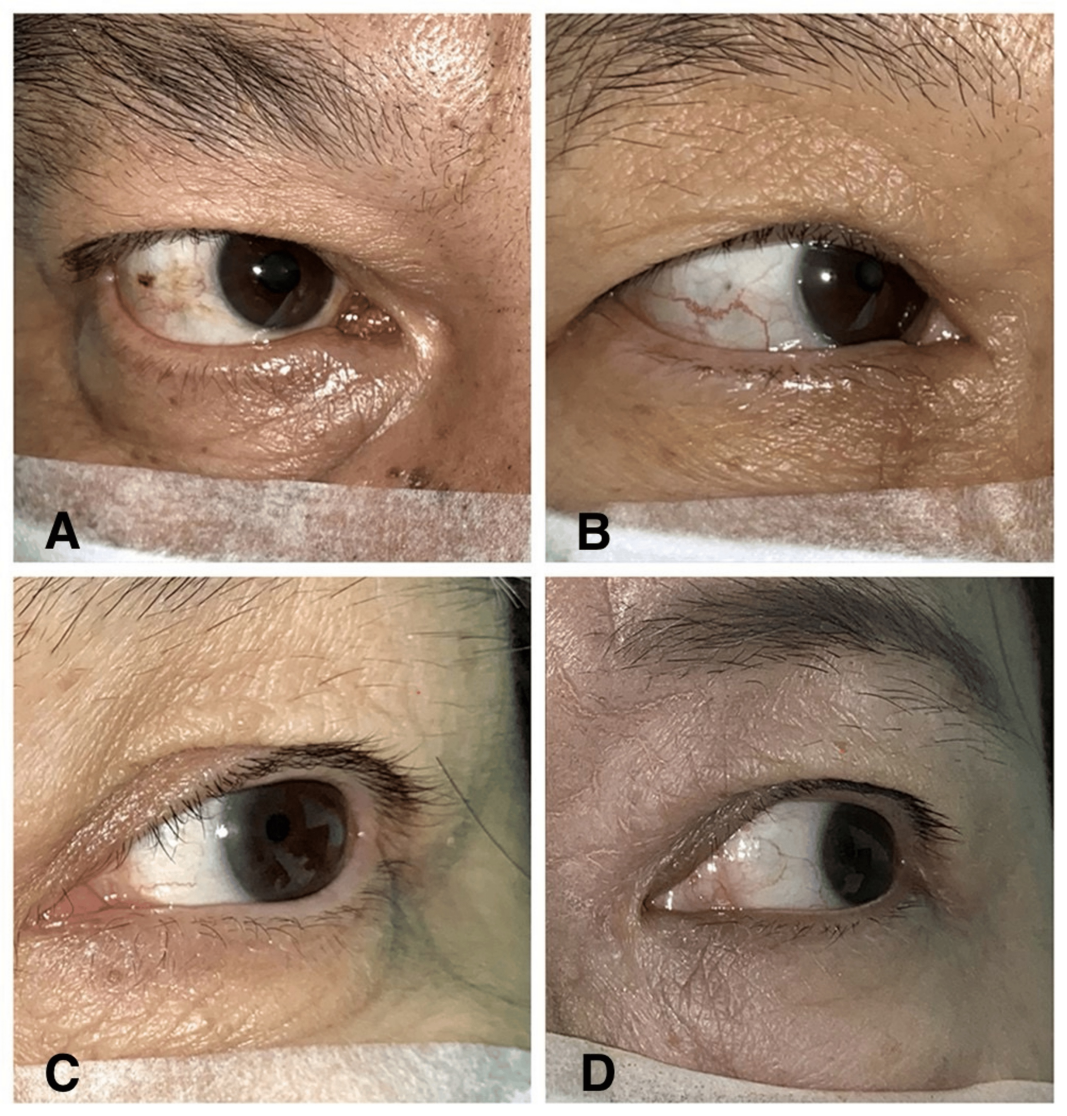
Ocular duction images illustrating each range of movement: 30 PD (A), 60 PD (B), 90 PD (C), and full duction pass the midline (D) PD: prism diopters

Captured images were systematically categorized to facilitate the training and evaluation of the CNN models, relying solely on the position of the corneal light reflex. In assessing the duction range, specific criteria were applied: When the corneal light reflex was positioned at the pupillary margin, the duction was classified as 30 PD beyond the midline. If the light reflex appeared halfway between the pupillary margin and the limbus, it was assigned a value of 60 PD from the midline. A light reflex at the limbus indicated 90 PD, while a corneal light reflex extending beyond the limbus onto the sclera was considered a full duction. These distinctions allowed for a clear and reproducible classification framework, as illustrated in Figure 2, which provides visual examples of the assigned categories.

A single strabismus specialist thoroughly evaluated each image to ensure consistency and accuracy. This specialist classified the images into four groups based on the precise positioning of the corneal light reflex, corresponding to the established duction ranges. Only images meeting the criteria for these four duction categories were utilized in the study. This approach enabled the CNN models to learn and interpret each specific range of ocular movement effectively. By limiting the dataset to these carefully selected subclasses, the models were trained, evaluated, and tested with a focus on accurately detecting the predefined ranges of ocular duction.

The transfer learning approach was strategically used in applying CNN to develop image analysis to test the range of duction movement. Leveraging pre-trained CNN models reduced image noise, thereby enhancing the accuracy and robustness of duction movement predictions.

Model selection

The study employed a CNN implemented through TensorFlow in Python, a widely used supervised deep learning framework known for its expertise in image classification tasks. The core objective was to train the CNN to detect and classify images of ocular duction accurately. Three specific CNN models, MobileNet-V3 [21], ResNet-101 [22], and EfficientNet-B5 [23], were chosen to analyze the duction positions, each bringing distinct strengths to the study.

In CNN-based image analysis, the network learns to identify key features in images through multiple layers of convolutional and pooling operations. In this task, the CNN models were trained using labeled images of eyes in different duction positions. This allowed them to identify patterns related to corneal light reflex deviations indicative of various duction ranges. The input images passed through a series of convolutional layers, where filters automatically detected features like edges, curves, and textures. The models refined their recognition through deeper layers, becoming progressively more sophisticated in identifying specific ocular positions. This hierarchy of feature extraction allowed CNN to differentiate subtle variation induction, thereby facilitating precise classification.

Each model demonstrated particular strengths suited to the challenge of ocular duction detection. MobileNet-V3, designed for efficiency, focuses on reducing computational demands without sacrificing accuracy, making it an excellent choice for real-time analysis with limited resources. Its streamlined architecture involves depthwise separable convolutions, which reduce the number of parameters while maintaining performance, optimizing it for mobile and embedded applications.

ResNet-101, by contrast, employs a deeper architecture, characterized by residual connections. These connections help avoid the vanishing gradient problem in deep networks, enabling ResNet-101 to capture complex patterns in high-dimensional data. This depth makes it particularly effective in identifying nuanced differences between images of varying ocular movements.

EfficientNet-B5 stands out for its innovative approach to balancing the three key factors in CNN architecture: depth, width, and resolution. Using a compound scaling strategy, EfficientNet-B5 optimizes these dimensions simultaneously, leading to a model that can handle large, high-resolution images with a higher level of detail. This model's scalability allows it to maintain high accuracy even as computational complexity increases, making it versatile for diverse imaging conditions.

By leveraging the unique strengths of each model, the study aimed to accurately detect ocular duction patterns while evaluating the trade-offs between computational efficiency and prediction accuracy. This comprehensive approach ensured that the CNNs could handle the diverse and subtle challenges associated with identifying different ranges of ocular movement, contributing valuable insights to clinical applications in ophthalmology.

Based on standard practices in AI modules, the selection adhered to a common heuristic of acquiring a substantial volume of data, typically around 1,000 images per class, wherein the minimum threshold frequently rests at a few hundred photos per class. This strategy ensures robust model performance and comprehensive learning across the various ocular duction categories.

Model validation and testing

The training process involved 50 complete iterations (epochs) during which the model adapted its internal parameters. This adaptation was guided by the Adam optimizer, with a learning rate set at 0.001 to adjust the model's learning pace. Cross-entropy, an important mathematical function measuring prediction accuracy against actual labels, was employed as a loss function to guide the model's learning process.

The testing data were evaluated by calculating and presenting the area under the receiver operating characteristic (AUROC) curve, a widely acknowledged metric for assessing model performance. To mitigate the risk of data leakage, a rigorous methodology was implemented, meticulously segregating the testing datasets from the training and validation datasets. This separation ensured the model's robustness and capacity to generalize unseen data, fortifying the credibility of the evaluation process.

Statistical analysis

The AUROC curve was utilized to gauge the models' performance by assessing their ability to differentiate between classes. This indicated the association between sensitivity (true positive rate) and 1-specificity (false positive rate) across varying threshold values. A higher AUROC curve value (0-1) indicated a superior discrimination ability. In the ocular duction measurement, AUROC curve values were calculated for different duction positions (30 PD, 60 PD, 90 PD, and full duction). These values were important indicators of the models' predictive prowess, thereby offering insights into their capability to accurately distinguish between distinct ocular duction positions based on their predictive performance.

One-way analysis of variance (ANOVA) was used to compare the mean AUROC curve across different models within four distinct subclasses. A p-value of <0.05 indicated statistically significant differences among the mean AUROC curve across these model subclasses.

## Results

Classification of participants

In total, 150 participants were excluded from the current study. The remaining 526 participants were enrolled in this study. Then, 2,404 images of participants were included in this analysis, thereby facilitating the training and internal validation of the CNN model. These images were categorized based on ocular movements into four sets: 30 PD, 60 PD, 90 PD, and the full range of ductions. Table 1 shows the number of images. However, 47 images were omitted from the analysis because of inadequate quality and deviation from the assigned ocular orientation.

**Table 1:** 2,404 images used for training, validating, and testing CNNs PD: prism diopters; CNNs: convolutional neural networks

	30 PD	60 PD	90 PD	Full
Training	310	437	216	1038
Validation	40	44	20	109
Testing	25	45	20	100

Performance of the deep learning model in classifying ocular duction in the evaluation phase

Table 2 depicts the comprehensive details outlining the model's performance metrics resulting from the training setup. The internal validation outcomes of the deep learning model included sensitivity, specificity, negative predictive value, and positive predictive value. The training and validation accuracies of MobileNet-V3 were 0.9139 and 0.8056, respectively. The training and validation accuracies of ResNet-101 were 0.7964 and 0.7778. The training and validation accuracies of EfficientNet-B5 were 0.8887 and 0.8056, respectively.

**Table 2:** Performance metric of each model in image processing artificial intelligence

Model	Image size (pixels)	Training accuracy	Validation accuracy	Training time (minutes)
MobileNet-V3	224	0.9139	0.8056	05:54
ResNet-101	224	0.7964	0.7778	09:56
EfficientNet-B5	224	0.8887	0.8056	26:39

Receiver operating characteristic (ROC) curve of deep learning for classifying ocular duction images in the testing phase

**Figure 3:**
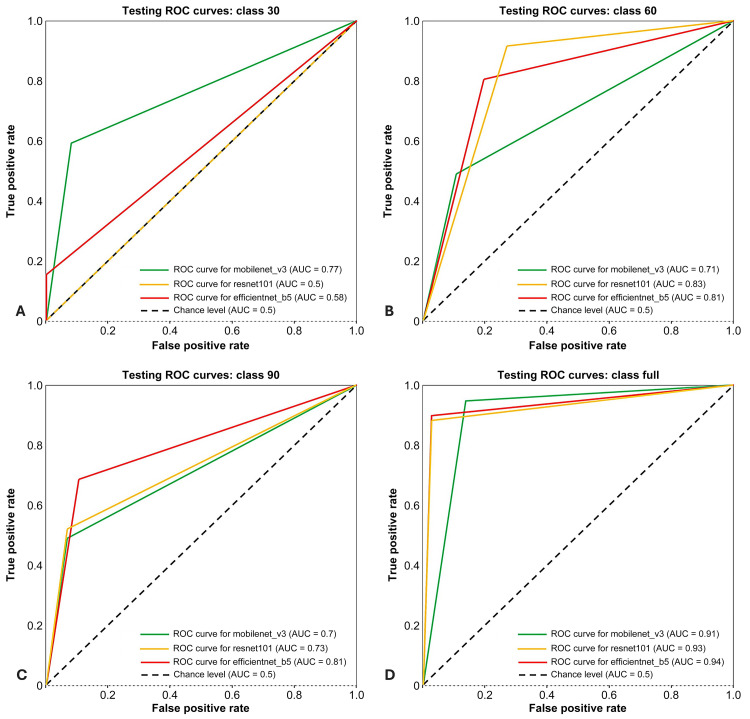
ROC curves of MobileNet_v3, ResNet101, and EfficientNet_B5 models in predicting ocular duction across testing dataset ROC curves comparing the performance of MobileNet_v3, ResNet101, and EfficientNet_B5 models in predicting ocular duction angles from the testing dataset. Panel A shows the ROC curve for 30 PD beyond the midline, Panel B for 60 PD beyond the midline, Panel C for 90 PD beyond the midline, and Panel D for full ocular duction beyond the midline. ROC: receiver operating characteristic; PD: prism diopters

In the subsequent test data, as presented in Figure 3, the assessment was conducted using all three CNN models, MobileNet_V3_Large, ResNet101, and EfficientNet_B5, to measure the ROC curves for various duction positions. The ROC curves of MobileNet_V3_Large, ResNet101, and EfficientNet_B5 were 0.77, 0.5, and 0.58 for 30 PD duction; 0.71, 0.83, and 0.81 for 60 PD duction; 0.7, 0.73, and 0.81 for 90 PD duction; and 0.91, 0.93, and 0.94 for full duction, respectively.

Differences between each model

**Figure 4:**
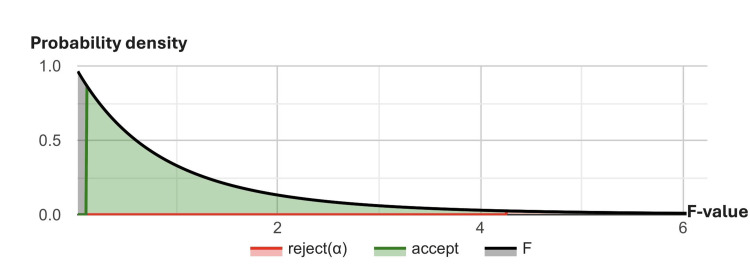
One-way ANOVA results comparing three CNN models in four ocular gaze directions ANOVA: analysis of variance; CNN: convolutional neural network

The one-way ANOVA indicated no statistically significant differences in AUROC curve performance among the three CNN models, MobileNet, ResNet, and EfficientNet, across the four gaze ranges of ocular duction, with a p-value of 0.936 (Table 3). Table 4 displays the confidence intervals for the mean AUROC curve of each model, with MobileNet showing slightly narrower intervals compared to ResNet and EfficientNet. Figure 4 illustrates the F-distribution for the ANOVA results, reflecting the nonsignificant variance in AUROC curve scores among the three models.

**Table 3:** One-way ANOVA of the mean AUROC curve ANOVA: analysis of variance; AUROC: area under the receiver operating characteristic; DF: degrees of freedom

	DF	Sum of squares	Mean square	F statistics	P-value
Between groups	2	0.002917	0.001458	0.06661	0.936
Within groups	9	0.1971	0.02189		
Total	11	0.2	0.01818		

**Table 4:** Confidence intervals for average prediction accuracy across three CNN models CNN: convolutional neural network

Model	Confidence interval (from-to)
MobileNet	0.5-1
ResNet	0.5-1.5
EfficientNet	0.5-1.5

## Discussion

The current study utilized three CNN models, MobileNet, ResNet, and EfficientNet, to evaluate ocular duction across four ranges, 30 PD, 60 PD, 90 PD, and full duction, crossing the midline in patients without histories of ocular surgery, ocular defects, strabismus, or cranial nerve disorders (III, IV, or VI). Each model was trained to classify these four duction ranges, achieving evaluation accuracies above 70% on validation data assigned by ophthalmologists. One-way ANOVA indicated no statistically significant differences in performance among the three models. MobileNet demonstrated narrower confidence intervals and faster learning times, suggesting potential practical advantages, particularly in settings where computational efficiency is prioritized. Despite a lack of statistical significance, these qualities may render it an optimal choice for clinical application, as they contribute to a consistent, efficient performance across duction ranges. MobileNet's streamlined architecture and reduced computational demands allow it to achieve swift training and deployment without sacrificing predictive accuracy, underscoring its practical utility for real-time ocular diagnostics.

In evaluating full duction, all three models surpassed 90% accuracy, potentially due to the larger volume of training data available for this specific duction range. This highlights the impact of data balance on model learning and performance, an important consideration for future research. In ophthalmology, AI is transforming diagnostic workflows, offering precise analysis of complex datasets like retinal images and visual field tests, which enhances efficiency, especially for diagnosing conditions like diabetic retinopathy and glaucoma [13,14]. Advanced frameworks such as TensorFlow [24] facilitate deep learning in detecting ocular movement ranges from photographic data. MobileNet's compact size suits real-time applications, while EfficientNet balances accuracy and computational load, and ResNet's deep residual connections enable complex feature extraction for precise ocular motion analysis. AI can support accurate ocular movement assessments by utilizing these models, potentially augmenting ophthalmic diagnostics and treatment planning.

Furthermore, recent studies, such as those by de Figueiredo et al. [16] and Lou et al. [17], underscore AI's role in ocular movement analysis. de Figueiredo et al. [16] used AI to classify nine gaze positions, emphasizing image standardization's importance and enhancing AI's diagnostic capacity for strabismus. Lou et al. [17] employed deep learning to assess ocular movement, finding solid correlations between AI analysis and manual measurements, as well as an inverse relationship between movement range and age. In our study, standardizing image capture was critical for model consistency, achieved by stabilizing the head position at a fixed distance with a consistent background frame. Such standardization minimizes image saturation and brightness variability, which is crucial for training AI models focused exclusively on eye position.

Future research should aim to expand these deep learning models' capabilities in ocular diagnostics, emphasizing real-time data integration and clinical data linkage. Enhancing real-time data analysis could provide immediate insights into ocular health, thereby advancing patient care and creating new research opportunities in ophthalmology. Integrating more balanced datasets across all duction ranges and establishing automated data flow into AI systems could further refine model performance. While our models provided valuable insights, they currently serve as complements to ophthalmologists rather than replacements, as human expertise remains essential for nuanced diagnoses and patient-centered care.

Limitations

This study has several limitations that should be considered when interpreting the findings. First, the data used to evaluate the three CNN models across four gaze positions were not collected from the same subject, precluding the application of repeated-measures ANOVA, which may have limited the robustness of statistical comparisons across models. Instead, one-way ANOVA was employed, which, while useful, does not account for within-subject variability that could offer a more nuanced understanding of model performance. Additionally, the uneven distribution of datasets across different ranges of duction affected model training and the interpretation of various ocular duction types, potentially impacting the accuracy and generalizability of the results. Addressing this imbalance with uniform data collection across the entire duction range would likely enhance the models' learning capabilities and their ability to interpret diverse ocular duction patterns.

Furthermore, the data collection process required manual data transfer, as the images did not directly flow into the AI system; implementing an online system for automated data transfer would streamline analysis and increase efficiency. This study also did not include participants with pathological conditions causing limited ocular duction, so the findings may not be generalizable to patients with such conditions. Although our results indicated that AI measurements of ocular duction were comparable to those of ophthalmologists, the models cannot replicate human clinical experience, adaptability, or a patient-centered approach. Human expertise remains essential for complex diagnoses, ethical considerations, and interdisciplinary insight, positioning AI as a complementary tool rather than a replacement in ophthalmic care.

## Conclusions

The three CNN models examined in this study did not significantly differ in terms of accuracy in detecting the four ocular duction ranges. However, based on our findings, MobileNet_v3 is the most optimal model among the three. Notably, our study underscored a robust correlation, surpassing 70%, between AI interpretation and assigned clinician measurements. This emphasizes that CNNs can be a promising tool for assisting ophthalmologists in duction measurement and interpretation.

Future directions involve refining models for improved accuracy in ocular duction prediction, a wider range of duction measurements, expanding datasets for comprehensive training, and exploring advanced deep learning techniques. These enhancements create user-friendly applications that help in clinical diagnosis and treatment.
